# Structure of *rrn* operons in pathogenic non-cultivable treponemes: sequence but not genomic position of intergenic spacers correlates with classification of *Treponema pallidum* and *Treponema paraluiscuniculi* strains

**DOI:** 10.1099/jmm.0.050658-0

**Published:** 2013-02

**Authors:** Darina Čejková, Marie Zobaníková, Petra Pospíšilová, Michal Strouhal, Lenka Mikalová, George M. Weinstock, David Šmajs

**Affiliations:** 1 Department of Biology, Faculty of Medicine, Masaryk University, Kamenice 5, Building A6, 62500 Brno, Czech Republic; 2 The Genome Institute, Washington University in St Louis, 4444 Forest Park Avenue, St Louis, MO 63108, USA

## Abstract

This study examined the sequences of the two rRNA (*rrn*) operons of pathogenic non-cultivable treponemes, comprising 11 strains of *T. pallidum* ssp. *pallidum* (TPA), five strains of *T. pallidum* ssp. *pertenue* (TPE), two strains of *T. pallidum* ssp. *endemicum* (TEN), a simian Fribourg-Blanc strain and a rabbit *T. paraluiscuniculi* (TPc) strain. PCR was used to determine the type of 16S–23S ribosomal intergenic spacers in the *rrn* operons from 30 clinical samples belonging to five different genotypes. When compared with the TPA strains, TPc Cuniculi A strain had a 17 bp deletion, and the TPE, TEN and Fribourg-Blanc isolates had a deletion of 33 bp. Other than these deletions, only 17 heterogeneous sites were found within the entire region (excluding the 16S–23S intergenic spacer region encoding tRNA-Ile or tRNA-Ala). The pattern of nucleotide changes in the *rrn* operons corresponded to the classification of treponemal strains, whilst two different *rrn* spacer patterns (Ile/Ala and Ala/Ile) appeared to be distributed randomly across species/subspecies classification, time and geographical source of the treponemal strains. It is suggested that the random distribution of tRNA genes is caused by reciprocal translocation between repetitive sequences mediated by a *recBCD*-like system.

## Introduction

rRNA genes are co-localized in rRNA (*rrn*) operons. The typical bacterial *rrn* operon consists of 16S–23S–5S rRNA genes. In addition, *rrn* operons may contain tRNA genes and regulatory regions. The *rrn* operons are highly transcribed in bacteria (Condon *et al.*, 1992), especially during the exponential phase of growth and in fast-growing bacteria. It is generally believed that bacteria with a short generation time have multiple *rrn* operons in the genome. Multiple copies of 16S and 23S rRNA genes in an organism are almost identical (Pei *et al.*, 2009, 2010), suggesting homogenization of rRNA genes through homologous recombination (Liao, 2000). The 16S and 23S rRNA genes are widely used in bacterial phylogenetic studies, but the 5S rRNA genes are too short to be useful for this purpose.

In addition to the rRNA genes, the *rrn* operons contain intergenic spacer regions (ISRs). The ISRs are not involved in ribosomal function, so they are not under functional constraints, resulting in higher ISR microheterogeneity among bacterial species and strains (de Vries *et al.*, 2006; Gürtler, 1999). The 16S–23S ISRs vary in length, tRNA composition and intragenomic nucleotide diversity (Stewart & Cavanaugh, 2007), and have been used for bacterial identification, molecular typing (Indra *et al.*, 2010; Sadeghifard *et al.*, 2006) and evolutionary studies (Antón *et al.*, 1998).

In this study, we used the variation present in the *rrn* operons to assess evolutionary relationships among several pathogenic non-cultivable treponemes. The organisms studied comprised *Treponema pallidum* and *Treponema paraluiscuniculi* species and an unclassified simian isolate (Fribourg-Blanc). The species of *T. pallidum* comprised *T. pallidum* ssp. *pallidum* (TPA), *T. pallidum* ssp. *pertenue* (TPE) and *T. pallidum* ssp. *endemicum* (TEN), the aetiological agents of syphilis, yaws and endemic syphilis, respectively. *T. paraluiscuniculi* (TPc), the aetiological agent of rabbit syphilis, and the simian Fribourg-Blanc isolate are closely related to the *T. pallidum* spp. (Šmajs *et al.*, 2011a).

Two *rrn* operons have been observed in pathogenic treponemes (Fukunaga *et al.*, 1992) composed of 16S–23S–5S rRNA genes. The 16S–23S ISRs of the TPA Nichols strain (Fraser *et al.*, 1998) contain tRNA-Ile (tRNA-Ile-1; TP_t12) and tRNA-Ala (tRNA-Ala-3; TP_t15) genes within the *rrn1* and *rrn2* operons, respectively. The same spacer pattern (Ile/Ala) has been observed in other complete treponemal genomes (Giacani *et al.*, 2010; Matějková *et al.*, 2008; Šmajs *et al.*, 2011b). In contrast, the TPE CDC-2 and TPE Gauthier strain genomes (Čejková *et al.*, 2012) show an Ala/Ile spacer pattern, where the TP_t12 and TP_t15 orthologues are located within the *rrn2* and *rrn1* operons, respectively.

Stamm *et al.* (2002) used the sequences of 16S–23S ISRs for molecular typing of dermatitis-associated treponemes in cattle. These treponemes are divided into three phylotypes, which cluster within the group of human saprophytic treponemes (*Treponema denticola*, *Treponema phagedenis* and *Treponema vincentii*). Centurion-Lara *et al.* (1996) examined the TPA Nichols and TPE Gauthier strains and found no difference in the 16S–23S ISRs.

Closely related spirochaetes in the genus *Borrelia* contain two distinct *rrn* operon patterns. Whereas Lyme disease agent (*Borrelia burgdorferi sensu lato*) harbours a unique operon composed of 16S–23S–5S–23S–5S rRNA genes, agents of relapsing fever carry an operon consisting of 16S–23S–5S rRNA genes (Fraser *et al.*, 1997; Schwartz *et al.*, 1992). Two typing systems have been developed using the 16S–23S ISR, which includes both the tRNA-Ala and tRNA-Ile genes (Bunikis *et al.*, 2004; Liveris *et al.*, 1996). The typing systems have been applied to differentiate species within *B. burgdorferi sensu lato* in North America (Bunikis *et al.*, 2004), to study populations of tick- and bird-borne *Borrelia garinii* in Eurasia (Comstedt *et al.*, 2009) and to study the association between the *B. burgdorferi sensu stricto* genotype and dissemination of infection (Hanincová *et al.*, 2008; Wormser *et al.*, 2008).

In this study, we compared the sequences of both *rrn* operons among pathogenic treponemes, comprising 11 strains of TPA, five strains of TPE, two strains of TEN, a simian Fribourg-Blanc isolate and a rabbit TPc strain. We also studied 16S–23S ISRs in 30 clinical samples positive for *T. pallidum* DNA.

## Methods

### 

#### Strains used in this study.

The *rrn* operon sequences were examined in 20 strains of the genus *Treponema* (Table 1), comprising a baboon isolate (unclassified *T. pallidum* strain Fribourg-Blanc), a rabbit syphilis strain (TPc) and 18 human strains (TPA, TPE and TEN). Thirty clinical samples (named 2K, 4K, 6K, 15K, 24K, 27K, 34K, 40K, 44K, 47K, 49K, 51K, 52K, 53K, 63K, 73K, 91K, 6000, 9888, 14048, 14207, 16142, RL86Z, RL89BZ, RL95B, RL102B, RL104B, RL110B, RL111B and RL116A) were tested for the presence of 16S–23S ISR sequences encoding either tRNA-Ile or tRNA-Ala, with positive detection of treponemal DNA in all samples. More detailed data on these samples were published recently (Flasarová *et al.*, 2012).

**Table 1.** *Treponema* strains used in this study –, Not known.

Strain	*Treponema* (sub)species	Place of isolation	Date of isolation	Reference	Source of material*
Bal 73-1	TPA	Baltimore, USA	1968	Hardy *et al.* (1970)	David L. Cox, CDC, Atlanta, GA, USA
Bosnia A	TEN	Bosnia	1950	Turner & Hollander (1957)	Sylvia M. Bruisten, PHL, Amsterdam, NL
CDC-1	TPE	Dersuso, Ghana	1980	Liska *et al.* (1982)	David L. Cox, CDC, Atlanta, GA, USA
CDC-2	TPE	Akorabo, Ghana	1980	Liska *et al.* (1982)	Steven J. Norris, UT, Houston, TX, USA
Cuniculi A	*paraluiscuniculi*	–	pre-1957	Turner & Hollander (1957)	Steven J. Norris, UT, Houston, TX, USA
DAL-1	TPA	Dallas, USA	1991	Wendel *et al.* (1991)	David L. Cox, CDC, Atlanta, GA, USA
Fribourg-Blanc	Simian isolate	Guinea	1966	Fribourg-Blanc & Mollaret (1969)	David L. Cox, CDC, Atlanta, GA, USA
Gauthier	TPE	Brazzaville, Congo	1960	Gastinel *et al.* (1963)	Steven J. Norris, UT, Houston, TX, USA
Grady	TPA	Atlanta, USA	1980s	–	David L. Cox, CDC, Atlanta, GA, USA
Haiti B	TPA	Haiti	1951	Turner & Hollander (1957)	David L. Cox, CDC, Atlanta, GA, USA
Iraq B	TEN	Iraq	1951	Turner & Hollander (1957)	Kristin N. Harper, Emory University, Atlanta, GA, USA
Madras	TPA	Madras, India	1954	Laboratory notebook of Rob George CDC	David L. Cox, CDC, Atlanta, GA, USA
Mexico A	TPA	Mexico City, Mexico	1953	Turner & Hollander (1957)	David L. Cox, CDC, Atlanta, GA, USA
MN-3	TPA	Minnesota, USA	–	–	David L. Cox, CDC, Atlanta, GA, USA
Nichols	TPA	Washington, DC, USA	1912	Nichols & Hough (1913)	Steven J. Norris, UT, Houston, TX, USA
Philadelphia 1	TPA	Philadelphia, USA	1988	–	David L. Cox, CDC, Atlanta, GA, USA
Philadelphia 2	TPA	Philadelphia, USA	–	–	David L. Cox, CDC, Atlanta, GA, USA
Samoa D	TPE	Samoa	1953	Turner & Hollander (1957)	Steven J. Norris, UT, Houston, TX, USA
Samoa F	TPE	Samoa	1953	Turner & Hollander (1957)	Steven J. Norris, UT, Houston, TX, USA
SS14	TPA	Atlanta, USA	1977	Stamm *et al.* (1983)	Steven J. Norris, UT, Houston, TX, USA

* CDC, Centers for Disease Control and Prevention; PHL, Public Health Laboratory; UT, University of Texas.

#### Isolation of treponemal DNA.

TPA Nichols and SS14, TPE Samoa D and CDC-2, and TPc Cuniculi A chromosomal DNA was prepared as described previously by Fraser *et al.* (1998) by extracting DNA from experimentally infected rabbits. Treponemes were purified by Hypaque gradient centrifugation (Baseman *et al.*, 1974). Because a high input of DNA was required for the sequencing approach, whole-genome amplification (WGA) (REPLI-g Midi kit; Qiagen) was performed for TPA Nichols DNA according to the manufacturer’s instructions. In addition, non-WGA DNAs from TPA Nichols and SS14, TPE Samoa D and CDC-2, and TPc Cuniculi A were used. The Philadelphia 1, Philadelphia 2, DAL-1, Mexico A, Bal 73-1, Grady, MN-3, Madras and Haiti B (TPA), CDC-1, CDC-2, Gauthier and Samoa F (TPE), Bosnia A and Iraq B (TEN), and Fribourg-Blanc (a simian *T. pallidum*) strains were obtained as rabbit testicular tissues containing treponemal cells. After brief centrifugation of the samples at 100 ***g*** for 5 min, the DNA enriched for bacterial cells was amplified using the REPLI-g Midi kit.

#### PCR amplification.

The primer pairs RNA1F (5′-GTGTGTGAGTCTGGCAGGAA-3′) and RNA1R (5′-TTATTGCTGTGCGCATCTTC-3′), and RNA2F (5′-ACAAGTGAGCGAAGCGTTTT-3′) and RNA2R (5′-CCAAGAGAGCTACCCGTCTG-3′), were used for amplification of the *rrn* operons from treponemal strains. These primer pairs produced extra-large PCR (XL-PCR) products of 5.85 and 5.92 kb, respectively. To obtain these XL-PCR amplicons, a GeneAmp XL PCR kit (Roche Molecular Systems) was used as described by Strouhal *et al.* (2007). XL-PCR products were purified using a QIAquick PCR Purification kit (Qiagen) or ExoSAP-IT kit (GE Healthcare) according to the manufacturer’s instructions.

#### DNA sequencing.

DNA sequencing of the XL-PCR products was carried out with a BigDye Terminator v3.1 Cycle Sequencing kit (Applied Biosystems) using a primer-walking approach. Additional internal oligonucleotide sequencing primers (see Table S1, available in JMM Online) were designed using Primer3 software (Rozen & Skaletsky, 2000). The lasergene program package (DNASTAR) was used to assemble the consensus sequences.

#### Phylogenetic analyses.

In addition to the *rrn* operons investigated in the 20 strains (Table 1), the *rrn* operons of TPA Chicago (GenBank accession no. CP001752; Giacani *et al.*, 2010) was included in the evolutionary analysis. Concatenated sequences of *rrn1* and *rrn2* operons (Table S2) were used for the construction of evolutionary trees using the neighbour-joining method (Saitou & Nei, 1987) in mega4 software (Tamura *et al.*, 2007). The bootstrap consensus trees were determined from 1000 bootstrap resamplings. Branches with <50 % bootstrap support were collapsed.

#### Detection of recombination.

To identify genomic rearrangements, *rrn* operons were analysed using the Recombination Detection Program package (version rdp3; Martin *et al.*, 2010). Four methods, including rdp, geneconv (Sawyer, 1989), MaxChi (Smith, 1992) and chimaera (Posada & Crandall, 2001), implemented in the rdp3 package, were applied using default settings.

#### Analysis of clinical specimens.

Skin and mucosal swabs were placed in a tube containing 1.5 ml sterile water and agitated for 5 min at room temperature. The swab was withdrawn and the supernatant was used for DNA isolation. Swab supernatant (0.2–0.4 ml) and whole blood (0.2–0.8 ml) were used for DNA isolation using a QIAamp DNA Mini kit (Qiagen) according to the manufacturer’s Blood and Body Fluid Spin Protocol. To detect the presence of treponemal DNA in swab and whole-blood samples, a diagnostic PCR assay amplifying five different *Treponema*-specific genes including *polA* (TP0105 locus), *tmp*C (TP0319), TP0136, TP0548 and the 23S rRNA gene was performed. Amplification and subsequent sequencing of TP0136, TP0548 and the 23S rRNA gene have been used, although not for diagnostic purposes, for molecular typing of treponemal strains (Flasarová *et al.*, 2006, 2012; Liu *et al.*, 2001; Matějková *et al.*, 2009; Woznicová *et al.*, 2007).

**Fig. 1.**
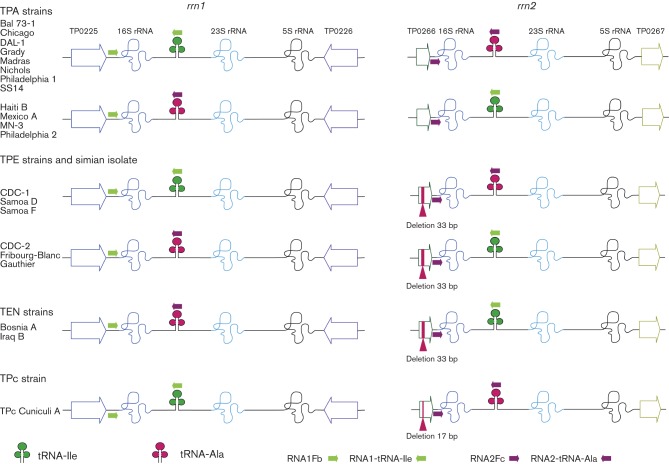
A schematic representation of the treponemal *rrn* operons consisting of 16S–23S–5S rRNA genes, intergenic regions and a 212 bp DNA sequence upstream of 16S rRNA gene. The positions of the 33 and 17 bp deletions in the non-TPA strains are shown. Please note that both spacer patterns of the 16S–23S ISR encoding either tRNA-Ile or tRNA-Ala were present among TPA and TPE strains in the *rrn1* and *rrn2* regions. Green symbols represent tRNA-Ile, whilst red symbols represent tRNA-Ala. Small coloured arrows in green and purple represent primers used in the clinical samples examined.

The composition of 16S–23S ISR sequences in the *rrn1* and *rrn2* operons, encoding either tRNA-Ile or tRNA-Ala, was determined by another nested PCR. In the first step, each clinical isolate was tested in four parallel reactions with the following primer pairs (Fig. 1 and Table S3): RNA1Fb and RNA1-tRNA-Ile (first reaction), RNA1Fb and RNA2-tRNA-Ala (second reaction), RNA2Fc and RNA1-tRNA-Ile (third reaction) and RNA2Fc and RNA2-tRNA-Ala (fourth reaction). Using these primer sets, the PCR products revealed the position (*rrn1* or *rrn2*) and composition (tRNA-Ile or tRNA-Ala) of the amplified *rrn* operon. In the second step of the nested PCR, the PCR product of the *rrn1* (from the first and second reactions) region was amplified using TP0225-6aF and TP0225-6bR primers, whilst the PCR product of the *rrn2* (from the third and fourth reactions) region was amplified with RNA2Fa and TP0225-6bR. The second step was not specific for the Ile/Ala or Ala/Ile *rrn* spacer pattern but improved the sensitivity of detection of the PCR product from the first step. Each PCR contained 0.4 µl 10 mM dNTP mix, 2 µl 10× ThermoPol Reaction buffer (New England BioLabs), 0.1 µl each primer (100 pmol µl^−1^), 0.1 µl *Taq* DNA polymerase (5000 U ml^−1^; New England BioLabs), 1 µl test sample and 16.3 µl PCR-grade water, giving 20 µl in total. PCR amplification was performed using a GeneAmp 9800 thermocycler (Applied Biosystems) with the following cycling conditions: 94 °C for 5 min; 40 cycles of 94 °C for 60 s, 72 °C for 20 s and 72 °C for 150 s; and a final extension at 72 °C for 10 min. The second step of the nested PCR used the same conditions but a lower annealing temperature of 67 °C.

## Results

### Amplification and sequencing of the *rrn* operons

Two *rrn* operons (16S–23S–5S) have been described in pathogenic *Treponema* genomes with the 16S–23S ISR comprising genes encoding tRNA-Ala or tRNA-Ile (Fraser *et al.*, 1998; Fukunaga *et al.*, 1992; Giacani *et al.*, 2010; Šmajs *et al.*, 2011b). Using XL-PCR, we amplified the *rrn* operons in 20 treponemal strains (Tables 1 and S2) comprising 11 strains of TPA, five strains of TPE, an unclassified simian isolate, two strains of TEN and a rabbit TPc isolate. XL-PCR products were obtained for all 40 investigated regions. However, the assembled sequence of the *rrn2* operon of Iraq B (TEN) was repeatedly ambiguous at several positions, probably due to low DNA quality, so the Iraq B sequences were excluded from the construction of phylogenetic trees.

### Sequence analysis of *rrn* operons

**Fig. 2.**
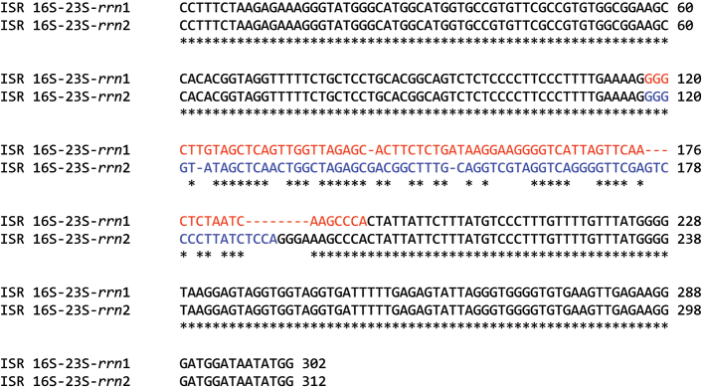
Alignment of 16S–23S ISRs in TPA Nichols *rrn* operons. The gene encoding tRNA-Ile (TP_t12) is shown in red, whilst the gene encoding tRNA-Ala-3 (TP_t15) is in blue.

In the individual TPA genomes, the amplified *rrn1* and *rrn2* regions were identical for 5141 bp (Tables 2 and S2, Fig. 1) including the DNA regions 212 bp upstream of the 16S rRNA, the 16S rRNA (1537 bp), 23S rRNA (2951 bp), 5S rRNA (110 bp) and 23S–5S ISR (50 bp), and a region of 54 bp downstream of the 5S rRNA. Additional identical sequences were located within the 16S–23S ISR downstream of the 16S rRNA (120 bp) and upstream of the 23S rRNA (118 bp) genes (Fig. 2, Table 2). Alternative sequences within the 16S–23S ISR, encoding tRNA-Ile or tRNA-Ala, comprised an additional 64 or 74 bp, respectively (Fig. 2). To extend the comparative analysis over all available data, the TPA Chicago sequences of the *rrn* operons (GenBank accession no. CP001752; Giacani *et al.*, 2010) were added to the sequences of the 20 strains used in this study.

**Table 2.** DNA sequence polymorphisms found among 20 pathogenic *Treponema* strains at the *rrn* operons SNPs are indicated by underlining, whereas translocation of tRNA is shown in bold. IGR, Intergenic region.

Species	Strain (operon)	Treponemal homologous sequences of rRNA operons, and position downstream (D), upstream (U) or within the RNA gene																		
		IGR (212 bp)			16S rRNA (1537 bp)				IGR (117 or 116 bp)*	tRNA (74 bp)*	IGR (111 or 122 bp)*	23S rRNA (2951 bp)							IGR (50 bp)	5S rRNA (110 bp)
		171–167 U	96 U	93 U	647	1134	1375	1441	71 D		21 U	458	763	766	1092	1359	1546	2104	47 D	81
TPA	Bal 73-1 (*rrn1*)	GGGGG	A	A	G	G	G	C	G	tRNA-Ile	G	G	G	G	G	A	A	A	C	C
	Bal 73-1 (*rrn2*)	GGGGG	A	A	G	G	G	C	G	tRNA-Ala	G	G	G	G	G	A	A	A	C	C
	Chicago (*rrn1*)	GGGGG	A	A	G	G	G	C	G	tRNA-Ile	G	G	G	G	G	A	A	A	C	C
	Chicago (*rrn2*)	GGGGG	A	A	G	G	G	C	G	tRNA-Ala	G	G	G	G	G	A	A	A	C	C
	DAL-1 (*rrn1*)	GGGG	A	A	G	G	G	C	G	tRNA-Ile	G	G	G	G	G	A	A	A	C	C
	DAL-1 (*rrn2*)	GGGGG	A	A	G	G	G	C	G	tRNA-Ala	G	G	G	G	G	A	A	A	C	C
	Grady (*rrn1*)	GGGGG	A	A	G	G	G	C	G	tRNA-Ile	G	G	G	G	G	A	A	A	C	C
	Grady (*rrn2*)	GGGGG	A	A	G	G	G	C	G	tRNA-Ala	G	G	G	G	G	A	A	A	C	C
	Haiti B (*rrn1*)	GGGGG	A	A	G	G	G	C	G	**tRNA-Ala**	G	G	G	G	G	A	A	A	C	C
	Haiti B (*rrn2*)	GGGGG	A	A	G	G	G	C	G	**tRNA-Ile**	G	G	G	G	G	A	A	A	C	C
	Madras (*rrn1*)	GGGGG	A	A	G	G	G	C	G	tRNA-Ile	G	G	G	G	G	A	A	A	C	C
	Madras (*rrn2*)	GGGGG	A	A	G	G	G	C	G	tRNA-Ala	G	G	G	G	G	A	A	A	C	C
	Mexico A (*rrn1*)	GGGGG	A	A	G	G	G	C	G	**tRNA-Ala**	G	G	G	G	G	A	A	A	C	C
	Mexico A (*rrn2*)	GGGGG	A	A	G	G	G	C	G	**tRNA-Ile**	G	G	G	G	G	A	A	A	C	C
	MN-3 (*rrn1*)	GGGGG	A	A	G	G	G	C	G	**tRNA-Ala**	G	G	G	G	G	A	A	A	C	C
	MN-3 (*rrn2*)	GGGGG	A	A	G	G	G	C	G	**tRNA-Ile**	G	G	G	G	G	A	A	A	C	C
	Nichols (*rrn1*)	GGGGG	A	A	G	G	G	C	G	tRNA-Ile	G	G	G	G	G	A	A	A	C	C
	Nichols (*rrn2*)	GGGGG	A	A	G	G	G	C	G	tRNA-Ala	G	G	G	G	G	A	A	A	C	C
	Philadelphia 1 (*rrn1*)	GGGGG	A	A	G	G	G	C	G	tRNA-Ile	G	G	G	G	G	A	A	A	C	C
	Philadelphia 1 (*rrn2*)	GGGGG	A	A	G	G	G	C	G	tRNA-Ala	G	G	G	G	G	A	A	A	C	C
	Philadelphia 2 (*rrn1*)	GGGGG	A	A	G	G	G	C	G	**tRNA-Ala**	G	G	G	G	G	A	A	A	C	C
	Philadelphia 2 (*rrn2*)	GGGGG	A	A	G	G	G	C	G	**tRNA-Ile**	G	G	G	G	G	A	A	A	C	C
	SS14 (*rrn1*)	GGGGG	A	A	G	G	G	C	G	tRNA-Ile	G	G	G	G	G	A	A	G	C	C
	SS14 (*rrn2*)	GGGGG	A	A	G	G	G	C	G	tRNA-Ala	G	G	G	G	G	A	A	G	C	C
TPE	CDC-1 (*rrn1*)	GGGGG	A	G	G	G	G	C	G	tRNA-Ile	G	G	G	A	G	A	A	A	C	C
	CDC-1 (*rrn2*)	GGGGG	A	G	G	G	G	C	G	tRNA-Ala	G	G	G	A	G	A	A	A	C	C
	CDC-2 (*rrn1*)	GGGGG	A	G	G	G	G	C	G	**tRNA-Ala**	G	G	G	A	G	A	A	A	C	C
	CDC-2 (*rrn2*)	GGGGG	A	G	G	G	G	C	G	**tRNA-Ile**	G	G	G	A	G	A	A	A	C	C
	Gauthier (*rrn1*)	GGGGG	A	G	G	G	G	C	G	**tRNA-Ala**	G	G	G	A	G	A	A	A	C	C
	Gauthier (*rrn2*)	GGGGG	A	G	G	G	G	C	G	**tRNA-Ile**	G	G	G	A	G	A	A	A	C	C
	Samoa D (*rrn1*)	GGGGG	A	G	G	G	G	C	G	tRNA-Ile	G	G	G	A	G	A	A	A	C	C
	Samoa D (*rrn2*)	GGGGG	A	G	G	G	G	C	G	tRNA-Ala	G	G	G	A	G	A	A	A	C	C
	Samoa F (*rrn1*)	GGGGG	A	G	G	G	G	C	G	tRNA-Ile	G	G	G	A	G	A	A	A	C	C
	Samoa F (*rrn2*)	GGGGG	A	G	G	G	G	C	G	tRNA-Ala	G	G	G	A	G	A	A	A	C	C
Simian isolate	Fribourg-Blanc (*rrn1*)	GGGGG	A	G	G	G	G	C	G	**tRNA-Ala**	G	A	G	A	G	A	A	A	C	C
	Fribourg-Blanc (*rrn2*)	GGGGG	A	G	G	G	G	C	G	**tRNA-Ile**	G	A	G	A	G	A	A	A	C	C
TEN	Bosnia A (*rrn1*)	GGGGG	A	A	G	G	A	C	G	**tRNA-Ala**	G	G	G	A	G	A	A	A	C	C
	Bosnia A (*rrn2*)	GGGGG	A	A	G	G	A	C	G	**tRNA-Ile**	G	G	G	A	G	A	A	A	C	C
	Iraq B (*rrn1*)	GGGGG	A	A	G	G	A	C	G	**tRNA-Ala**	G	G	G	A	G	A	A	A	C	C
	Iraq B (*rrn2*)	GGGGG	A	A	G	G	A	C	G	**tRNA-Ile**	G	G	G	A	G	A	A	A	C	C
TPc	Cuniculi A (*rrn1*)	GGGGG	G	A	A	A	G	T	A	tRNA-Ile	A	G	A	A	A	G	G	A	T	A
	Cuniculi A (*rrn2*)	GGGGG	G	A	A	A	G	T	A	tRNA-Ala	A	G	A	A	A	G	G	A	T	A

* The size of sequence between the 16S and 23S rRNA genes (both excluded) varied based on the presence of the tRNA-Ile (117+74+111, in total 302 bp) or tRNA-Ala (116+74+122, in total 312 bp) gene.

When compared with the TPA strains, a deletion of 33 bp was found in homologous regions of the *rrn2* region in the TPE, TEN and simian strains (Fig. 1), whilst the TPc strain contained a 17 bp deletion at the same position (Fig. 1). These deletions resulted in shortening (33 bp deletion) or truncation (17 bp deletion) of TP0266 orthologues. Among all investigated strains, in addition to the observed deletions, we found only 17 heterogeneous sites within the entire region, excluding the 16S–23S ISR encoding tRNA-Ile or tRNA-Ala. Sixteen sites were single nucleotide changes and one was a single base-pair deletion (Table 2). The *rrn1* operon of the reference TPA Nichols genome (GenBank accession no. AE000520.1; Fraser *et al.*, 1998) showed a deletion within the 16S rRNA gene (data not shown), whereas all other strains, including the Nichols strain examined in our study, did not. This deletion may represent a sequencing error present in the reference Nichols genome, as dozens of such sequencing errors have already been confirmed (Giacani *et al.*, 2012; Matějková *et al.*, 2008). In contrast, a 1 bp deletion in the TPA DAL-1 genome, upstream of the 16S rRNA gene in the *rrn1* operon, was repeatedly confirmed by Sanger sequencing. The identified nucleotide change at position 2104 of the 23S rRNA gene (differentiating the SS14 strains from other investigated strains) corresponded to the mutation causing macrolide resistance in treponemal strains (Stamm & Bergen, 2000).

All TPA strains differed from the other pathogenic treponemes by a nucleotide change at position 766 of the 23S rRNA gene. The TPE strains and the simian isolate Fribourg-Blanc could be distinguished from the other pathogenic treponemes by a single-nucleotide polymorphism (SNP) localized 93 bp upstream of the 16S rRNA genes. The TPE strains could be differentiated from the simian isolate by a nucleotide sequence change in the 23S rRNA gene (nt 458). The TEN showed a nucleotide change in the 16S rRNA gene, and TPc showed 12 nt changes in the investigated *rrn* sequences (Table 2).

### Reciprocal translocation of tRNA genes

In contrast to the phylogenetically conserved SNP distribution in the repetitive sequences of the *rrn* operons, the genes coding for tRNA did not show the same evolutionary pattern (Table 2, Fig. 1). In this study, we observed two 16S–23S ribosomal ISR patterns. The spacer pattern Ile/Ala included the tRNA-Ile gene within the *rrn1* region and the tRNA-Ala gene within the *rrn2* region. The Ile/Ala pattern was observed in the following strains: TPA Nichols, Bal 73-1, Grady, SS14, Chicago, DAL-1, Philadelphia 1 and Madras; TPE Samoa D, CDC-1 and Samoa F; and TPc Cuniculi A. The reverse ISR pattern Ala/Ile consisted of the tRNA-Ala gene within the *rrn1* region and the tRNA-Ile gene within the *rrn2* region. The Ala/Ile pattern was found in TPA Mexico A, MN-3, Philadelphia 2 and Haiti B strains; in TPE Gauthier and CDC-2; in the unclassified treponeme Fribourg-Blanc; and in TEN Iraq B and Bosnia A genomes.

**Fig. 3.**
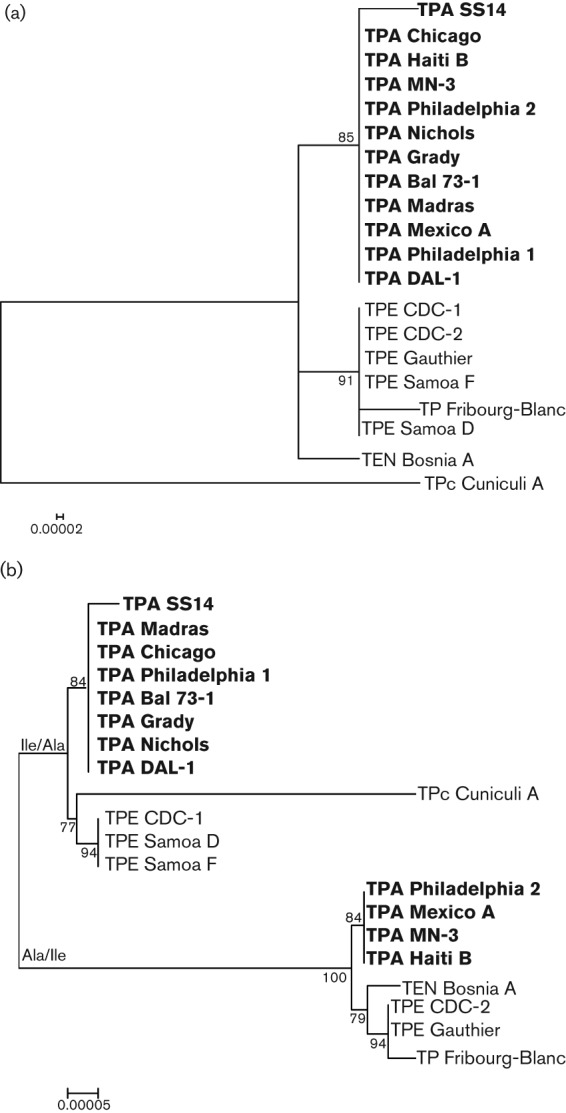
(a) An unrooted tree constructed from the concatenated sequences of the *rrn* operons excluding the heterologous tRNA genes. The *rrn* operons clustered according to the species/subspecies classification of treponemes. Bar, 0.00002 nt substitutions per site. (b) An unrooted tree constructed from the sequences of the *rrn* operons including heterologous tRNA genes. The *rrn* operons clustered according to the ISR pattern. Bar, 0.00005 nt substitutions per site. Bootstrap values based on 1000 replications are shown next to the branches. The TPA strains causing syphilis are shown in bold.

The concatenated *rrn* operons, excluding the tRNA genes and their vicinity, clustered according to the species/subspecies classification (Fig. 3a). The TEN Iraq B strain was omitted from the analysis because we were unable to obtain an unambiguous *rrn2* operon sequence. Nevertheless, the *rrn1* operon was identical to another TEN strain, Bosnia A. In contrast, the trees showing concatenated *rrn* operons including tRNA genes (Fig. 3b) branched according to the composition of tRNA in the individual *rrn* operons, and then according to the species/subspecies classification. This phenomenon can be explained by recombination events that have occurred between *rrn* operons.

To predict recombination hot-spot sites within the *rrn* operons, four methods from the rdp3 program were applied. All four methods predicted four recombination sites (Table 3), two sites in each *rrn* operon. The predicted sites corresponded to the same positions within the 16S (nt 783) and 23S (nt 324) rRNA genes in both *rrn* operons.

**Table 3.** Predicted recombination hot-spot sites using the rdp3 program

Predicted recombination hot-spot site*		Prediction algorithm used in the rdp3 program (*P* value)			
Start	End	RDP	geneconv	MaxChi	chimaera
231656	233036	1.33E−58	2.20E−55	8.06E−13	7.80E−13
280058	281448	7.22E−20	1.12E−20	5.91E−04	1.33E−03

* Whole-genome TPE Samoa D coordinates are shown (Čejková *et al.*, 2012; GenBank accession no. CP002374).

### Structure of the *rrn* operons in clinical isolates containing *T. pallidum* DNA

The composition of the 16S–23S ribosomal ISR (Ile/Ala or Ala/Ile spacer pattern in the *rrn* operons) was tested in 30 recently isolated clinical samples (Flasarová *et al.*, 2012). The results are summarized in Table 4. Only the Ile/Ala pattern was identified in all clinical samples tested, despite the fact that the clinical samples belonged to five different genotypes (Table 4), as revealed by CDC and sequencing-based typing (Flasarová *et al.* 2012; Pillay *et al.* 1998). Nevertheless, all clinical strain genotypes were similar to the SS14 strain genotype.

**Table 4.** Composition of the 16S–23S ribosomal ISR in clinical samples containing TPA DNA

Clinical sample	16S–23S ISR (*rrn1*/*rrn2*)	Genotype*	Subtype†
2K	Ile/Ala	SU2R8	14d
4K	Ile/Ala	SSS	14d
6K	Ile/Ala	SSS	14d
15K	Ile/Ala	SSS	14d
24K	Ile/Ala	SSS	14d
27K	Ile/Ala	SSS	14d
34K	Ile/Ala	SSS	14d
40K	Ile/Ala	SSR8	14d
44K	Ile/Ala	SSS	14d
47K	Ile/Ala	SSS	12d
49K	Ile/Ala	SSR8	14d
51K	Ile/Ala	SSS	14d
52K	Ile/Ala	SSS	14d
53K	Ile/Ala	SSS	14d
63K	Ile/Ala	SSR9	15d
73K	Ile/Ala	SU2R8	14d
91K	Ile/Ala	SSS	14d
6000	Ile/Ala	SU2R8	14d
9888	Ile/Ala	SSS	14d
14048	Ile/Ala	SSS	14d
14207	Ile/Ala	SSS	14d
16142	Ile/Ala	SU2R8	14d
RL86Z	Ile/Ala	SU2R8	14d
RL89BZ	Ile/Ala	SSS	14d
RL95B	Ile/Ala	SSS	14d
RL102B	Ile/Ala	SSS	14d
RL104B	Ile/Ala	SU2R8	14d
RL110B	Ile/Ala	SSS	14d
RL111B	Ile/Ala	SU2R8	14d
RL116A	Ile/Ala	XXR8	14e

* Identified according to the method of Flasarová *et al.* (2012).

† Subtype according to the method of Pillay *et al.* (1998).

## Discussion

In this study, we examined the *rrn* operons in 20 pathogenic treponemal strains and 30 clinical isolates. All investigated strains contained two copies of the *rrn* operons. Two *rrn* operons with the same composition have also been described in other human and animal treponemes except for *T. vincentii* containing only one *rrn* operon (Fraser *et al.*, 1998; Matějková *et al.*, 2008; Seshadri *et al.*, 2004; Stamm *et al.*, 2002).

Our results confirmed that there is little diversity within rRNA genes and ISRs. However, our data showed that the *rrn* operon structure displayed blocks of conserved and polymorphic sites. The TPA DAL-1 strain showed a 1 bp deletion upstream of the 16S rRNA gene in the *rrn1* operon. It is known that TPA DAL-1 grows more rapidly in rabbits than other pathogenic strains (Wendel *et al.*, 1991), and it is possible that the different promoter DNA conformation may affect expression of the *rrn1* operon.

Gürtler & Stanisich (1996) used the 16S–23S ribosomal ISR for classification of bacteria. 16S–23S ISRs have been used in several studies (de Vries *et al.*, 2006; Lan & Reeves, 1998; Lebuhn *et al.*, 2006), including for treponemal (Centurion-Lara *et al.*, 1996; Stamm *et al.*, 2002) and borrelian samples (Bunikis *et al.*, 2004; Comstedt *et al.*, 2009). Centurion-Lara *et al.* (1996) examined the TPA Nichols and TPE Gauthier strains, and no difference was found. However, they did not examine the genomic positions of individual 16S–23S ISRs. Interestingly, the 16S–23S ISR typing of *Borrelia burgdorferi sensu stricto* is in accordance with *ospC* gene typing (Hanincová *et al.*, 2008; Wormser *et al.*, 2008). The *ospC* gene, encoding a protein involved in the initiation of infection in warm-blooded animals, is located on plasmid DNA, whilst the *rrn* operon is on chromosomal DNA. Moreover, different 16S–23S ISR genotypes are associated with different degrees of invasivity (Wormser *et al.*, 2008).

Despite the low heterogeneity in the *rrn* operons, two different ISR patterns were observed in the pathogenic treponemal samples. Whereas detection of specific nucleotide changes may be of interest in identification of treponemal diseases, the detection of tRNA genes in the 16S–23S ribosomal ISR appears to be of limited use in typing of clinical samples. All clinical samples showed the Ile/Ala spacer pattern in *rrn* operons, so the tRNA-Ile and tRNA-Ala genes are not useful for molecular typing of clinical strains, at least for treponemes present in the population of the Czech Republic.

Due to the conserved machinery of protein synthesis, rRNA genes are expected to be under strong purifying selection and are exposed to the intragenomic homogenization process via gene conversion (Liao, 2000; Nei & Rooney, 2005). Several studies (Acinas *et al.*, 2004; Pei *et al.*, 2009, 2010) have shown that homogenization of multiple rRNA genes is common among bacteria. In addition, Harvey & Hill (1990) successfully constructed several *Escherichia coli* strains with recombined inverted *rrn* operons; however, the recombinants tended to recover the original configuration. The *rrn* operons of treponemal strains are direct repeats: the tRNA-Ala gene is replaced by tRNA-Ile (and vice versa), and the recombination is a common event with no correlation to the otherwise-determined phylogenetic relationship among tested treponemes. It has been postulated that recombination between direct repeats leads to the duplication or deletion of a repeat (Petes & Hill, 1988; Petit, 2005). Whereas tRNA-Ile (TP_t12) is a unique gene in sequenced treponemal genomes, there are three predicted tRNA-Ala genes (TP_t15, TP_t41 and TP_t45; Fraser *et al.*, 1998). As both tRNA-Ile (TP_t12, GenBank accession no. AE000520.1) and tRNA-Ala (TP_t15, AE000520.1) genes need to be maintained in the genomes of pathogenic treponemes, reciprocal translocation, rather than gene conversion, appears to be the mechanism for the observed *rrn* heterogenity among tested strains. Such a process would require double cross-overs in both *rrn* operons, and therefore is much less common than insertion/deletion or gene-conversion events (Harvey & Hill, 1990; Hashimoto *et al.*, 2003). Predicted recombination hot-spot sites were located in the 16S and 23S rRNA genes, genes with two identical copies within every strain examined in our study.

During replication of direct-repeat regions, DNA polymerase might lead to strand slippage, thus collapsing a replication fork formation, and recombination enzymes are involved in the DNA repair machinery (Darling *et al.*, 2008; Santoyo & Romero, 2005). Although only the *recF* recombination pathway was predicted in the TPA Nichols genome (Fraser *et al.*, 1998), the *recF* pathway suggests the gene-conversion mechanism (Kobayashi, 1992; Takahashi *et al.*, 1992). Therefore, the reciprocal recombination in pathogenic treponemes may be accompanied by crossing-over, a repair mechanism implemented by the *recBCD* pathway in *E. coli* (Kobayashi, 1992). Recently, *recBCD* orthologues (*addA* and *addB*) were predicted for several investigated treponemal genomes (Čejková *et al.*, 2012; Giacani *et al.*, 2012; Šmajs *et al.*, 2011b), composed of TP0898 and fused TP0899–TP0900 orthologues. However, it would be extremely difficult to prove experimentally the *recBCD*-mediated crossing-over mechanism in *T. pallidum.*

In summary, two different *rrn* spacer patterns (Ile/Ala and Ala/Ile) seem to be distributed randomly across the time and place of original isolation of treponemal strains (e.g. Philadelphia 1 vs Philadelphia 2, CDC-1 vs CDC-2) and the laboratory that provided the treponemal material (Tables 1 and 2). This random distribution of tRNA genes is probably caused by reciprocal translocation between repetitive sequences mediated by a *recBCD*-like system.
